# Assessment of Ecological Risk of Heavy Metals Using Probabilistic Risk Assessment Model (AQUARISK) in Surface Sediments from Wami Estuary, Tanzania

**DOI:** 10.1155/2021/6635903

**Published:** 2021-07-09

**Authors:** Shovi Furaeli Sawe, Daniel Abel Shilla, John Ferdinand Machiwa

**Affiliations:** ^1^Tanzania Atomic Energy Commission, P O Box, 743 Arusha, Tanzania; ^2^Department of Aquatic Sciences, University of Dar es Salaam, P O Box, 35064 Dar es Salaam, Tanzania

## Abstract

Total concentrations of As, Cd, Cr, Cu, Pb, and Zn in sediment samples obtained from Wami Estuary in Tanzania were used to generate contaminant probability density distributions and species sensitivity distributions using the AQUARISK model. Results of tier 1 assessment showed that As, Cd, Cr, Pb, and Zn were not of concern as their measured values and the 99^th^ percentile of the fitted distributions were lower than the SQG low-trigger values. However, Cu was identified as of concern in this estuary. According to the Bur III distributional analysis of the exotoxicological data, the estimated percentage of species likely to be affected is 3.4, 79.4, 79.8, 99.9, 98.4, and 98.0 for As, Cd, Cr, Cu, Pb, and Zn, respectively. Lowering of the current median concentrations of metals (Cd, Cr, Cu, Pb, and Zn) is recommended as they exceeded modeled median target sediment concentration to achieve 95% or higher for species protection. With the ongoing increase in anthropogenic activities in the Wami River catchment, the environmental regulatory bodies may use the findings of the present study and augmented with AQUARISK to set discharge standards for various contaminants in order to minimize impacts to the receiving ecosystems.

## 1. Introduction

Human activities such as mining and agriculture are important sources of livelihood for modern societies in Tanzania. However, these activities lead to generation and release of potentially harmful substances such as heavy metals, pesticides, and hydrocarbons which end up in the aquatic environment [1]. Some metals dissolved in water are readily absorbed by fish and other aquatic organisms, and they can be toxic even at small concentrations because they undergo bioaccumulation. Metal toxicity can produce adverse effects to an organism's survival, growth, metabolism, or reproduction [2]. Runoff loaded with metals and other pollutants discharging into coastal waters from land-based activities can alter or degrade natural habitat [3, 4] and may lead to decline in aquatic biodiversity. In this regard, there are economic, ecological, and social concerns on the degradation of the coastal systems [5]. In addition to the aforementioned effects of aquatic pollution, agricultural crops irrigated with water from polluted water bodies have been reported to contain elevated levels of pollutants such as heavy metals [6, 7] and pesticides [8]. Concerns on human health due to consumption of foodstuffs with elevated levels of pollutants are therefore rising.

In response to these concerns, international organizations and regional and national authorities have established quality guidelines for water, sediments, and food, to help in understanding and protecting aquatic ecosystems and humans (see for example, Australian interim sediment quality guideline values for selected metals described in Twining et al. 2008 [9]; screening quick reference tables (SquiRTs) [10]; compilation of legal limits for hazardous substance in fish and fishery products [11]). Various risk assessment models have also been developed in order to assist national authorities in decision-making. One of such models is the probabilistic risk assessment model, AQUARISK. The AQUARISK model was developed by Australian Nuclear Science and Technology Organization (ANSTO) for quantitative probabilistic ecological risk assessments. The software uses the tiered approach in ecological risk assessment on aquatic ecosystems. The use of the tired approach in ecological risk assessment (ERA) is known to be one of the most effective ways to conduct ecotoxicological risk assessment in aquatic ecosystems [12–15]. The software (AQUARISK) can perform a probabilistic assessment of the risk for any metal by utilizing ecotoxicity or dose-response data [15, 16]. Subsequent tiers are termed as the detailed-level risk assessment [1]. With AQUARISK, estimates of the hazardous concentrations of metals in sediments or water likely to affect a certain percent of species at a chosen confidence level can be made. Also, the percentage of species at risk at a given metal concentration can be estimated with any level of confidence [9]. AQUARISK can also estimate both the degree to which the pollutant data are likely to exceed quality guideline levels and the required reduction in the median pollutant concentrations to achieve acceptable ecological risk. AQUARISK has been successfully used in Ghana to assess risk of metals in estuarine sediments [15, 17]. AQUARISK has also been used in Australia to perform probabilistic risk assessment due to metals in sediment from Sydney Harbour embayment [9].

In Tanzania, studies have shown that sediments from rivers discharging into the Indian Ocean are polluted with heavy metals originating from industrial, urban, and agricultural sources [7, 18–20]. This created the need to assess the ecological risk due to five metals and one metalloid in sediments from Wami Estuary. The aim was to estimate the probability of adverse events from current concentrations of heavy metals in sediments using the AQUARISK model. In this study, we have used species sensitivity distributions (SSDs) to establish cause–effect relationships between sediments As, Cd, Cr, Cu, Pb, and Zn and toxicity [21]. Similarly, probability density distributions (PDDs) of data acquired in this study have been compiled and compared with the interim sediment quality guideline values [9, 17]. We have estimated the hazardous concentration (HC) affecting *n*% of species at *p*% lower confidence limit (HC_*n*; *p*_), the percentage of species likely to be adversely affected by the contaminant concentrations, and the required reduction in the contaminant concentration and median target concentration to achieve acceptable risk. Results obtained from a probabilistic risk assessment model, AQUARISK, are presented and discussed.

## 2. Materials and Methods

### 2.1. Description of Study Site

**Figure 1:**
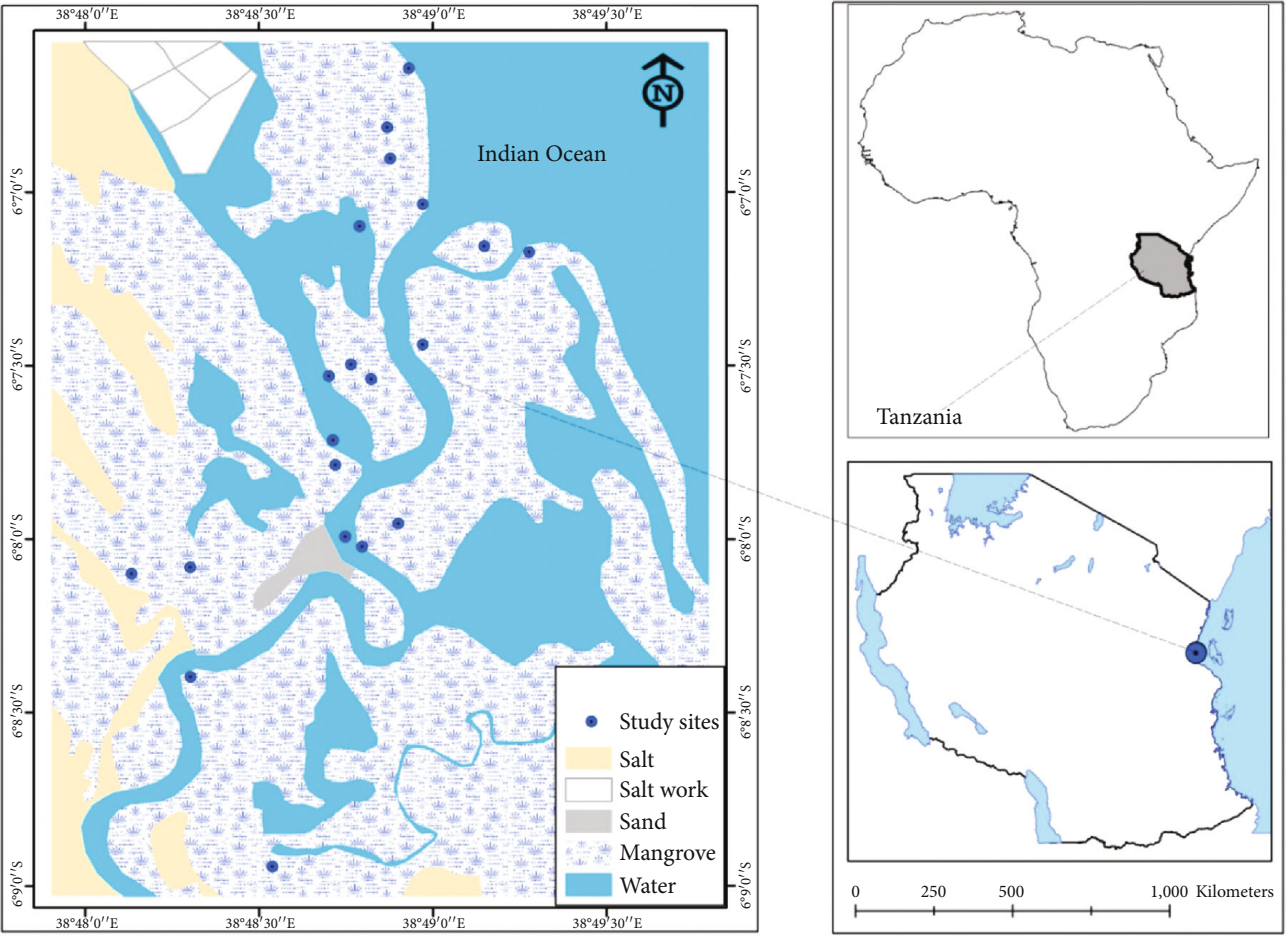
Schematic illustration of sampling points in the Wami Estuary, Tanzania.

Wami River in Tanzania rises from the Kaguru Mountains and flows in a south-east direction discharging into the Indian Ocean in Bagamoyo District [22]. The Wami Estuary, shown in Figure 1, is one of the most productive areas of Tanzania [23, 24]. The first five kilometers of the estuary are occupied by mangroves which serve as breeding and nursery grounds for fish, prawns, and birds [25]. The estuary also supports terrestrial wildlife by providing drinking water at its freshwater zone at the tidal limit during the dry season when other water sources in the area are dry. It also provides a habitat for mammals, crustaceans, reptiles, and birds [26]. The estuary receives runoff from industrial and residential areas as well as from sugarcane and rice plantations, among several other sources located in the catchment. The Wami subbasin comprises one of the world's most important hotspots of biological diversity: the Eastern Arc Mountains and coastal forests [22, 27]. The Estuary is known for its dense mangrove forests which have high metal retention capacity as reported elsewhere [20]. Since mangroves are recognized for providing shelters and breeding sites for marine juvenile fish [28], the accumulation of metals in the sediments may affect the well-being of faunal community.

### 2.2. Field Sampling

The judgmental sampling approach was adopted to ensure that the results obtained are reasonably representative of most habitats that are likely to exist in the study area. We considered both vegetated and unvegetated areas of the estuary. Samples were taken from upstream near the tidal limit, close to the sea, shallow sites that might be refuge sites for juveniles, deep areas that are likely habitats for large species, and mudflats that were assumed to be areas of high bacterial and phytoplankton productivity.

Short sediment cores were collected from 20 locations using hand driven PVC tubes. The uppermost 5 cm of sediment core was sliced and used for heavy metals analysis. Three samples were collected at each sampling station then pooled to form a composite representative sample for the station. Sediment samples were packed in ziplock plastic bags and placed in a cool box for transportation to the laboratory where they were kept frozen at -18°C in a freezer until were ready for analysis.

### 2.3. Sample Preparation and Analysis

Sediment samples were dried at room temperature for 12 hours then transferred into a drying oven at 55–60°C until constant weight was attained. Dried sediment samples were ground or segregated using pestle and mortar, and the resulting powder was sieved on a 63 *μ*m nylon mesh. Sediment fraction with grain size less than 63 *μ*m was then placed in plastic ziplock bags or desiccators to avoid absorption of moisture as described elsewhere [29]. Sediment subsamples (4 grams) of grain size less than 63 *μ*m were mixed with 0.9 grams of cellulose binder (FLUXANA®), homogenized in a pulverizer, and lastly pressed into a pellet of approximately 32 mm diameter. The obtained pellet was placed in the sample chamber of energy dispersive X-ray fluorescence (EDXRF) spectrometer (Spectro Xepos Model XEP01) for the determination of total elemental concentrations. Procedures for instrumental calibration and determination of elemental concentrations can be accessed in Rousseau et al. 1996 [30]. Three replicates from a composite sample for each location were analyzed for total elemental concentrations.

Analytical accuracy of the instrument and quality control was achieved by analyzing certified reference material (CRM) IAEA Soil 7 described in IAEA, 2000 [31]. Analytical values of elemental concentrations in the CRM were compared with the certified elemental concentrations and are shown in Table 1. The analytical concentration values agreed well with the certified, and the accuracy of the results was better than 97%.

### 2.4. Compilation of a Database and Probabilistic Ecological Risk Assessment

Elemental concentrations in sediment obtained during this study were compiled into a database. Formatting of the database was done according to AQUARISK User Guide and Technical Reference Manual. Moreover, the AQUARISK model requires the use of toxicity data for metals in order to estimate the percentage of affected species and the percentage reduction required in metal concentrations to achieve a given average percent of species affected. Since marine sediment toxicity (DRD) data hardly exists for Tanzania, we used DRD data from other areas for our tier 2 and 3 analyses. The data used in Twining et al. 2008 [9] to assess risk in Sydney harbor sediments formed the basis of data set used in this study.

Australian Interim Sediment Quality Guidelines (ISQG-Low) for elements presented in Table 2 were used in AQUARISK (user defined) for probabilistic ecological risk assessment. Procedures and assumption underlying the use of AQUARISK can be accessed from Twining et al. 2005 [32] and Twining et al. 2008 [9]. The first stage involved screening the results using AQUARISK by comparing metal concentrations with Interim Sediment Quality Guidelines (ISQG-Low). This stage was used to evaluate whether the emissions or discharges can put the receptor ecosystems at risk or not based on the sediment or water quality guideline. A more detailed probabilistic analysis was then performed on each metal by fitting cumulative probability density functions using log-normal and Burr type III distributions [33, 34] to both the concentration and effect data. The Kolmogorov-Smirnov test was used to assess the goodness-of-fit of the derived PDDs. Once the distribution parameters and their uncertainties were evaluated, critical values were also derived from the log-normal or Burr Type III SSDs for comparison with the ISQGs. These values were the median hazardous concentration (HC) affecting *n*% of species at 50% lower confidence limit (HC_*n*; 50_) and the 95% lower confidence limit (HC_*n*; 95_).

AQUARISK estimated the degree to which the contaminant data are likely to exceed the ISQG values and the critical values determined from the SSD. AQUARISK was used to convolute the two distributions (i.e*.,* PDD and SSD) for each element to determine the probability and extent that overlaps occur. This evaluated the percentage of species likely to be adversely affected by the contaminant concentrations.

Finally, the required reduction in the median contaminant concentrations to achieve acceptable risk was estimated using AQUARISK. This was in terms of the exceedance probability of the various criteria as well as the percentage of biotic species likely to be affected [9]. Hazardous concentrations of metals in sediments, the percentage of species likely to be adversely affected by the concentrations of metals in sediment, and the reductions and median target concentrations required to achieve no more than 5%, 10%, and 25% species impact due to that element were estimated using AQUARISK at 50 and 95% confidence levels for comparison.

### 2.5. Statistical Analyses

Mean concentrations ± standard deviations (SD) in mg/kg dry weight (dw) were determined using Excel spread sheet. Statistical analyses were performed using Statview 5 software. Pearson's correlation analysis between pairs of heavy metal concentrations was used to assess whether heavy metals in sediments had a common origin or not. According to Cynthia et al. [35] and Ra et al. [36], metals showing significant correlation might be originating from the same source. Significant differences were judged at a probability level of *p* < 0.05.

## 3. Results and Discussion

### 3.1. Total Elemental Concentrations

The mean and standard deviation (SD) of total elemental concentrations in sediment samples obtained during this study are presented in Table 3. The mean concentrations were used in AQUARISK to estimate hazardous concentrations (HC) of metals and percentage of species likely to be affected and target concentrations. Pearson's correlation analysis between pairs of heavy metal concentrations revealed that there was a significant positive correlation (*p* < 0.05) between the concentrations of Cu and Zn (*r* = 0.65) which may suggest common origin of these metals. The results showed that there was no significant correlation (*p* > 0.05) between Cu and Cd (*r* = 0.19), As and Pb (*r* = 0.07), Cd and Zn (*r* = 0.14), and Cu and Pb (*r* = 0.26) suggesting different sources of these metals. Furthermore, nonsignificant negative correlation observed for As and Cd (*r* = −0.05), As and Cr (*r* = −0.42), As and Zn (*r* = −0.46), and Cr and Cu (*r* = −0.24) (Table 4) suggests that these metals do not have common source as described elsewhere [35, 36]. Possible sources of heavy metals in the study area include agricultural land, mining areas, and weathering of metal-bearing rocks, among others.

### 3.2. Comparison of Elemental Concentrations with Sediment Quality Guidelines

**Figure 2:**
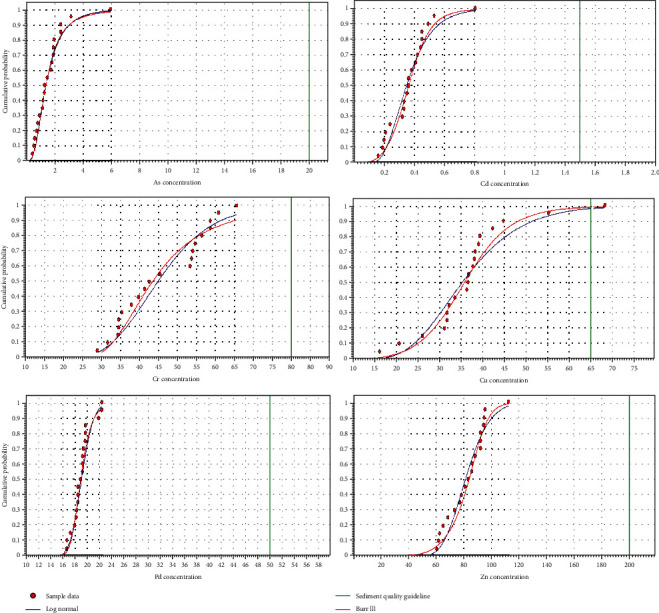
Fitted cumulative probability distribution of sediment concentrations of total As, Cd, Cr, Cu, Pb, and Zn (mg/kg) in the Wami Estuary using log-normal (blue) and Burr type III (red) functions. The vertical green line indicates the sediment quality guideline for each element.

Screening results showed that the ISQG-Low (Table 2) was exceeded by Cu only, and the ISQG-High was not exceeded by any element. The cumulative probability distributions for As, Cd, Cr, Cu, Pb, and Zn concentrations in sediments were generated by AQUARISK and are shown in Figure 2.

### 3.3. Hazardous Concentrations of Elements in Sediments

AQUARISK estimates of the hazardous concentrations of elements in sediments (ngg^−1^ dw) likely to affect up to 5, 10, or 25% of species at 50 and 95% confidence limit are presented in Table 5. Presented in columns two and three of Table 5 are the elemental concentrations estimated to be hazardous to 5% of species or 95% species protection at 50 and 95% confidence limit for columns two and three, respectively. Columns four and five contain elemental concentration estimated to be hazardous to 10% of species at 50% and 95% confidence limits for columns four and five, respectively. Similarly, columns six and seven contain metal concentration estimated to be hazardous to 25% of species or 75% species protection at 50% and 95% confidence limits for columns six and seven, respectively. The hazardous concentrations of metals in sediments changed considerably with the percentage of species protection and the chosen confidence level. For example, the hazardous concentrations of cadmium likely to affect up to 5% of species, i.e., 95% species protection (50% confidence limit) was 1.72 ngg^−1^ (column 2) but the concentration increased to 2.91 ngg^−1^ (column 4) when 10% of species were considered, i.e., 90% species protection at the same confidence limit. If we continue fixing the confidence limit at 50% and change the level of species protection, it can be seen that the hazardous concentration of cadmium likely to affect up to 5, 10, and 25% is 1.72, 2.91, and 8.14 ngg^−1^ for 95, 90, and 75% species protection, respectively. It can also be seen that the hazardous concentration of cadmium likely to affect up to 5% of species at 50% confidence limit was 1.72 ngg^−1^ but the concentration decreased to 0.19 ngg^−1^ when 95% confidence limit was considered at the same level of species protection. The same trend was observed for other elements.

### 3.4. Percentage of Species Likely to Be Affected and Target Concentrations of Elements

Estimates of species (%) likely to be affected by the current levels of As, Cd, Cr, Cu, Pb, and Zn; the hazardous concentrations of metals in sediments; and the required reduction of concentration (%) and median target concentration (ngg^−1^) for each element to achieve up to 5%, 10%, or 25% species effect at 50 and 95% confidence level were generated by AQUARISK (Tables 5 and 6). The median target concentrations for each element to achieve up to 5% at 95% confidence level (HC_5; 95_) is discussed. According to Table 5, AQUARISK results illustrate that the percentage of species likely to be affected by the existing concentrations of arsenic is 3.4, and no reduction in arsenic concentration required to meet the HC_5; 95_ criteria. In comparison, the percentage of species likely to be affected by the existing concentrations of cadmium is 79.4, and the percent reduction in Cd concentration required to meet the HC_5, 95_ criteria is 99.8 with target concentrations of 0.8 ngg^−1^. The current concentration of chromium is likely to affect up to 79.8% of species, and the required reduction in concentration is 99.9% with target concentration of 15.1 ngg^−1^. Likewise, the current level of copper is likely to affect up to 99.9% of species, and the required reduction is 99.9% to meet a target concentration of 2.6 ngg^−1^. The current concentration of lead is likely to affect up to 98.4% of species, and the required reduction is 99.8% to meet a target concentration of 46.2 ngg^−1^. Further, results show that the current concentration of zinc in the estuary is likely to affect up to 98% of species and therefore, a reduction of 99.9% is required to reach a target concentration of 15.0 ngg^−1^. The percentage of species likely to be affected by the current concentrations of metals in the estuary is the same regardless of the level of protection chosen (seen in columns 2-4 of Table 6). It can also be seen that the required reduction in concentration (%) decreases as the level of species protection decreases (see columns 5-7). Consequently, target concentrations increase as the percentage of species affected increases (see columns 8–10 of Table 6).

Wami Estuary is an important biological hot spot and therefore, the need to assess the ecological risk due to elements in sediments was realized. Comparison of elemental concentrations with Sediment Quality Guidelines (tier 1 assessment) revealed that As, Cd, Cr, Pb, and Zn were not of potential concern because all their measured values and the 99^th^ percentile of the fitted distributions were lower than the ISQG low-trigger values (Figure 2). However, Cu is identified to be of potential concern in this area because ISQG-Low was exceeded in one station. Sources of Cu in Wami Estuary include runoff from agricultural fields, industries, and small-scale mining practices taking place in the Wami River catchment. Figure 2 suggests that two distributions of Cr data may be possible implying that there are two different sources (presumably two different inflows) giving rise to this pattern.

AQUARISK estimates suggest that the percentage of species likely to be affected by the current levels of metals in sediments of Wami is high. For example, the percentage of species likely to be affected by As, Cd, Cr, Cu, Pb, and Zn is3.4, 79.4, 79.8, 99.9, 98.4, and 98.0, respectively, assuming species protection level of 95% at 95% confidence level (HC_5; 95_). The levels of percentage reduction required to meet the (HC_5; 95_) criteria were 0, 99.8, 99.9, 99.9, 99.8, and 99.9 for As, Cd, Cr, Cu, Pb, and Zn, respectively.

In comparison, a study conducted in Ghana by Mahu et al. 2014 [15] using AQUARISK showed that the concentrations of As in sediments of Ankobra Estuary was likely to affect 79% of species for the (HC_5; 95_) criteria, and the reduction required to meet the (HC_5; 95_) was estimated to be 99.9%. The percentage of species to be affected by As in Wami Estuary is far below that of Ankobra Estuary in Ghana, and no reduction in As concentration is required in Wami Estuary in order to meet the same criteria. Mahu et al. 2014 [17] showed that the concentrations of Cd in sediments of Sakumo II Estuary were likely to affect 77.6% of species for the (HC_5; 95_) criteria, and the reduction required to meet (HC_5; 95_) criteria was 100% while Twining et al. 2008 [9] showed that no reduction in cadmium concentration was required to achieve the (HC_5; 95_) criteria. The concentrations of Cu in the sediments of Sakumo II Estuary reported by Mahu et al. 2014 [15] were likely to affect 97.8% of species for the (HC_5; 95_) criteria, and the estimated percent reduction in Cu level required to meet the (HC_5; 95_) criteria was 100%. Twining et al. 2008 [9] showed that the reduction required to achieve (HC_5; 95_) criteria for copper was 94, 64, 80, 91, 90, and 88% for Hen and Chicken Bay, Homebush Bay—ASM, Homebush Bay—TM, Iron Cove, Long Bay, and Rozelle Bay, respectively. Mahu et al. 2014 [15] showed that the concentrations of Pb in Sakumo II Estuary were likely to affect 99.3% for the (HC_5; 95_) criteria, and the reduction required to meet the (HC_5; 95_) criteria was estimated to be 100%. The percentage of species to be affected by Pb in Wami Estuary is lower than that of Sakumo II Estuary in Ghana and consequently, the reduction required for Wami is lower than that of Sakumo II Estuary in order to meet the same criteria. Depending upon the level of protection placed on various estuaries, various trigger values may be chosen for management purposes. Higher levels of species protection result in lower levels of tolerable sediment contamination and vice versa as shown in Table 6. Different levels of species impact may be chosen depending upon the type of ecosystem being assessed. For example, a relatively pristine system that is home to rare or endangered species would warrant more restrictive levels of protection against contamination than a general marine reserve, national park, and an industrialized port. In addition, various levels of confidence can be applied depending on the amount of risk the site regulators are willing to accept when managing such sites.

## 4. Conclusion

The probabilistic risk assessment model, AQUARISK, was used to assess the ecological risk of heavy metals in sediments of Wami Estuary in Tanzania. Results of the screening exercise showed that the Interim Sediment Quality Guidelines-High (ISQG-High) was not exceeded by any heavy metals. Analysis of results showed that the hazardous concentrations of heavy metals in sediments changed considerably with the percentage of species protection and the chosen confidence level, and therefore, environmental managers who wish to use AQUARISK in setting waste water discharge standards or assessing ecological risk must choose these parameters carefully. The study further revealed that the percentage of species likely to be affected by the concentrations of measured elements followed the order Cu > Pb > Zn > Cr > Cd > As based on the Burr III distributional analysis of ecotoxicology data. We conclude further that the current median concentration of Cd, Cr, Cu, Pb, and Zn needs to be decreased as they exceed modeled median target sediment concentration to achieve 95% species protection or higher. With the ongoing increase in agricultural activities, mining, changing pattern of urbanization and industrialization, and in the catchment, environmental regulatory authorities may use the findings of this study augmented with AQUARISK to set discharge standards for various contaminants in order to reduce impacts to the receiving ecosystems.

## Figures and Tables

**Table 1:** Experimental values (mg kg^−1^) of As, Cd, Cr, Cu, Pb, Zn, and Fe compared with certified values in IAEA–SOIL–7 (trace elements in soil).

Element	Recommended value (mg kg^−1^)	95% confidence interval (mg kg^−1^)	Experimental value (mg kg^−1^)
As	13.4	12.5–14.2	13.6 ± 0.5
Cd	1.3	1.1–2.7	2.1 ± 0.2
Cr	60	49–74	58.9 ± 2.2
Cu	11	9–13	10.9 ± 1.6
Pb	60	55–71	58.6 ± 1.5
Zn	104	101–113	107 ± 4.5
Fe	25700	25200–26300	25708 ± 221

**Table 2:** Australian interim sediment quality guideline values (mg/kg dry weight) for selected metals.

Metal	As	Cd	Cu	Cr	Pb	Zn
ISQG-Low	20	1.5	65	80	50	200
ISQG-High	72	10	270	370	220	200

**Table 3:** Total elemental concentrations (mean ± SD) in sediment.

Location	Elemental concentration (mg kg^−1^dw)					
	As	Cd	Cr	Cu	Pb	Zn (s)
1	1.23 ± 0.06	0.45 ± 0.03	34.64 ± 2.70	37.96 ± 2.34	19.24 ± 1.36	68.72 ± 3.79
2	1.12 ± 0.08	0.36 ± 0.04	50.32 ± 3.43	36.6 ± 3.29	19.80 ± 1.36	77.2 ± 5.82
3	0.57 ± 0.04	0.80 ± 0.05	65.61 ± 4.64	44.73 ± 2.75	19.69 ± 2.08	95.24 ± 2.16
4	1.87 ± 0.10	0.36 ± 0.04	34.45 ± 2.44	36.67 ± 2.42	18.93 ± 0.83	62.07 ± 4.00
5	0.90 ± 0.07	0.32 ± 0.06	37.93 ± 5.36	26.07 ± 1.01	18.50 ± 2.11	62.57 ± 9.29
6	3.14 ± 0.25	0.53 ± 0.05	31.74 ± 3.03	32.21 ± 2.59	18.17 ± 1.29	65.17 ± 4.17
7	1.70 ± 0.13	0.32 ± 0.02	54.65 ± 8.12	37.68 ± 3.72	18.52 ± 1.70	78.42 ± 7.21
8	0.40 ± 0.05	0.45 ± 0.03	60.88 ± 4.30	39.3 ± 3.42	16.68 ± 1.41	85.8 ± 7.28
9	1.10 ± 0.09	0.35 ± 0.04	53.38 ± 4.91	39.02 ± 3.71	18.52 ± 1.57	92.15 ± 7.83
10	0.70 ± 0.03	0.49 ± 0.02	39.78 ± 3.38	55.14 ± 5.00	21.78 ± 0.98	112.38 ± 1.96
11	0.52 ± 0.01	0.44 ± 0.03	53.78 ± 4.56	16.28 ± 1.72	19.18 ± 1.44	94.75 ± 2.48
12	2.40 ± 0.07	0.36 ± 0.05	58.74 ± 5.20	31.71 ± 2.95	22.33 ± 2.21	92.11 ± 9.12
13	2.33 ± 0.27	0.41 ± 0.02	58.74 ± 6.15	31.71 ± 2.16	21.42 ± 1.58	92.11 ± 6.50
14	1.43 ± 0.15	0.38 ± 0.03	42.70 ± 4.22	20.82 ± 1.18	16.77 ± 1.30	88.25 ± 6.87
15	5.92 ± 0.12	0.42 ± 0.04	28.98 ± 2.05	68.14 ± 3.67	19.32 ± 1.91	60.74 ± 6.02
16	0.77 ± 0.05	0.21 ± 0.02	45.60 ± 3.56	42.28 ± 2.33	19.67 ± 1.67	86.05 ± 7.30
17	1.76 ± 0.07	0.24 ± 0.01	54.10 ± 5.35	31.12 ± 1.92	17.30 ± 1.22	83.16 ± 5.89
18	1.87 ± 0.15	0.16 ± 0.01	56.5 ± 4.80	36.32 ± 2.63	18.32 ± 1.68	94.35 ± 8.68
19	1.13 ± 0.08	0.20 ± 0.01	35.71 ± 2.52	33.6 ± 3.18	19.71 ± 1.95	73.5 ± 7.28
20	1.94 ± 0.12	0.19 ± 0.01	41.38 ± 3.79	38.2 ± 2.61	18.02 ± 1.28	81.05 ± 5.74

**Table 4:** Pearson's correlation coefficients between heavy metals are sediments.

	As	Cd	Cr	Cu	Pb	Zn
As	1.00					
Cd	-0.05	1.00				
Cr	-0.42	0.16	1.00			
Cu	0.46	0.19	-0.24	1.00		
Pb	0.07	0.18	0.07	0.26	1.00	
Zn	-0.46	0.14	0.65^∗^	-0.04	0.32	1.00

^∗^Correlation is significant at the 0.05 level (2-tailed).

**Table 5:** AQUARISK estimates of the hazardous concentrations of metals in sediments (ngg^−1^) likely to affect up to 5, 10, or 25% of species (at 50% and 95% confidence limit).

Element	Hazardous concentrations of metals in sediments likely to affect up to 5, 10 or 25% of species					
	HC5, 50	HC5, 95	HC10, 50	HC10, 95	HC25, 50	HC25, 95
As	3070	853.00	4660	2300	10900	4790
Cd	1.72	0.19	2.91	1.35	8.14	4.11
Cr	1.60	0.96	21.3	15.9	576	39.80
Cu	4.88	1.96	7.73	4.03	18.70	9.85
Pb	43.40	1.18	86.2	40.70	352.00	94.00
Zn	22.20	15.7	45.3	36.80	149.00	12.60

**Table 6:** AQUARISK estimates of species (%) likely to be affected by metals in sediment of Wami and the reductions required and median target concentrations required to achieve up to 5,10, or 25% species impact due to individual metal (95% confidence level).

Element	% of species affected			Required reduction (%)			Median target concentration (ngg^−1^)		
	HC_5_	HC_10_	HC_25_	HC_5_	HC_10_	HC_25_	HC_5_	HC_10_	HC_25_
As	3.4	3.4	3.4	NIL	NIL	NIL	1618.7	3066.4	8918.4
Cd	79.4	79.4	79.4	99.8	99.4	97.4	0.8	2.0	8.8
Cr	79.8	79.8	79.8	99.9	99.7	99.2	15.1	48.9	346.7
Cu	99.9	99.9	99.9	99.9	99.9	99.8	2.6	5.6	19.4
Pb	98.4	98.4	98.4	99.8	99.6	98.8	46.2	82.9	220.1
Zn	98.0	98.0	98.0	99.9	99.8	98.5	15.0	35.0	143.2

## Data Availability

Data for this manuscript have been included in this manuscript.
